# Determination of somatic oncogenic mutations linked to target-based therapies using MassARRAY technology

**DOI:** 10.18632/oncotarget.8002

**Published:** 2016-03-09

**Authors:** Maider Ibarrola-Villava, Tania Fleitas, Marta J. Llorca-Cardeñosa, Cristina Mongort, Elisa Alonso, Samuel Navarro, Octavio Burgues, Ana Vivancos, Juan Miguel Cejalvo, José Alejandro Perez-Fidalgo, Susana Roselló, Gloria Ribas, Andrés Cervantes

**Affiliations:** ^1^ Hematology and Medical Oncology Unit, Biomedical Research Institute INCLIVA, Valencia, Spain; ^2^ Department of Pathology, Biomedical Research Institute INCLIVA, Valencia, Spain; ^3^ Cancer Genomics Group, Vall d'Hebron Institute of Oncology (VHIO), Barcelona, Spain; ^4^ Hematology and Medical Oncology Unit, Clinic University Hospital of Valencia, Valencia, Spain; ^5^ Department of Medicine, University of Valencia, Valencia, Spain

**Keywords:** somatic oncogene mutations, personalized medicine, oncocarta

## Abstract

Somatic mutation analysis represents a useful tool in selecting personalized therapy. The aim of our study was to determine the presence of common genetic events affecting actionable oncogenes using a MassARRAY technology in patients with advanced solid tumors who were potential candidates for target-based therapies. The analysis of 238 mutations across 19 oncogenes was performed in 197 formalin-fixed paraffin-embedded samples of different tumors using the OncoCarta Panel v1.0 (Sequenom Hamburg, Germany). Of the 197 specimens, 97 (49.2%) presented at least one mutation. Forty-nine different oncogenic mutations in 16 genes were detected. Mutations in *KRAS* and *PIK3CA* were detected in 40/97 (41.2%) and 30/97 (30.9%) patients respectively. Thirty-one patients (32.0%) had mutations in two genes, 20 of them (64.5%) initially diagnosed with colorectal cancer. The co-occurrence of mutation involved mainly *KRAS*, *PIK3CA*, *KIT* and *RET*. Mutation profiles were validated using a customized panel and the Junior Next-Generation Sequencing technology (GS-Junior 454, Roche). Twenty-eight patients participated in early clinical trials or received specific treatments according to the molecular characterization (28.0%). MassARRAY technology is a rapid and effective method for identifying key cancer-driving mutations across a large number of samples, which allows for a more appropriate selection for personalized therapies.

## INTRODUCTION

Cancer is a complex group of diseases with many possible causes. It can be partly explain as a result of a progressive accumulation of mutations in cellular DNA, which provides a selective growth advantage to cancer cells and facilitates metastasis. Hotspot mutations are frequently present within oncogenes while some other aberrations are found in tumor suppressor genes in common solid tumors. The deregulation of certain signaling pathways, together with chromosomal abnormalities, has been identified in different solid tumors. Different oncogenic events have been described in cancer including mainly mutations in the RAS/RAF/MAPK and the PIK3/PTEN/AKT pathways. Therefore, mutations affecting the coding sequences of these specific genes are the hallmark of the disease and are currently targeted in clinical trials [1].

Our knowledge of cancer genomics has been enabled by the genome sequencing and other high-throughput omics technologies, leading to the discovery of new targets [2]. The development of targeted drugs has allowed for a more precise and personalized therapy, something which could be of major benefit to the patients. This drug sensitivity approach is reinforced by the efficacy shown in clinical trials using epidermal growth factor receptor (EGFR) and BRAF tyrosine kinase inhibitors (TKIs) [3–6]. The discovery of activating mutations located in the tyrosine kinase domains of EGFR has expanded the therapeutic options of lung cancer patients since they can be treated by EGFR-TKIs [7]. In metastatic colorectal cancer (mCRC) patients whose tumors are wild type for all KRAS/NRAS alleles, the administration of monoclonal antibodies against EGFR, such as cetuximab and panitumumab, in combination with conventional chemotherapy, substantially improves survival [8–10]. The presence of *KRAS* and *NRAS* mutations acts as a negative predictor to sensitivity to anti-EGFR monoclonal antibody therapy and, therefore, has caused an important change in the treatment of mCRC. The presence of the *BRAF* V600E activating mutation, found in approximately half of the diagnosed melanomas, is a turning point in the treatment of the metastatic disease through BRAF-TIKs [3, 11]. The use of targeted drugs against the oncogenic alterations of the *KRAS* gene and/or its downstream components (e.g. BRAF, MEK) seems to be a promising approach to cancer therapeutics either alone or in combination with other targeted agents [12–14].

Somatic mutation analysis has become a useful tool in selecting personalized therapies for many solid tumors. Mutation profiling can assist in the prognosis, prediction and treatment of solid tumors. Thus, molecular stratification for genotype-directed therapy could be required [15]. The mass spectrometry technique, matrix-assisted laser desorption/ionization-time of flight, has been used to assess point mutations across different solid tumors [16]. The Sequenom MassARRAY technology, in combination with a commercial kit called OncoCarta v1.0, was used to screen 238 somatic mutations across 19 oncogenes. This mutation panel interrogates somatic changes in oncogenes with known responses or resistance-targeted therapy. Custom assays can be also incorporated into the whole design, permitting the detection of specific target genes.

The goal of this study was to characterize the presence of common somatic mutations affecting known oncogenes in resected solid tumors that could provide potential therapeutic targets.

## RESULTS

### Patient characteristics

The median age of the patients was 58 years. The study included individuals with advanced-stage tumors who had received at least one line of treatment (67.4%).

The different tumor types representing the 197 enrolled patients were colorectal cancer (n= 75), breast cancer (n=73), ovarian cancer (n=10), lung cancer (n=9, 8 adenocarcinoma and 1 squamous), endometrial cancer (n=8) and other tumor types (n=20), including cervical, gastric, pancreatic, melanoma, anal, appendiceal, esophageal, renal, oral cavity and thyroid tumors. Formalin-fixed paraffin-embedded (FFPE) primary tumor samples were obtained for 123 (62.4%) subjects with nodal and/or metastatic tumor samples being available for a further 73 (37.1%) patients. The clinical characteristics of the patients have been included in Table 1. Colorectal and breast carcinoma were the two most represented tumor types with 75 and 73 cases enrolled, respectively (Supplementary Table S1).

**Table 1:** Classification of the samples studied by age and clinical characteristics (N=197)

Clinical characteristic	N (%)
**Age (years)**	
Median (Range)	58 (27-88)
**Gender**	
Female	129 (65.5)
Male	68 (34.5)
**Tumor type**	
Colorectal cancer	75 (38.1)
Breast cancer	73 (37.1)
Ovarian cancer	10 (5.1)
Lung cancer	9 (4.6)
Endometrial cancer	8 (4.0)
Others*	20 (10.1)
Unknown	2 (1.0)
**Prior therapy**	
No treatment	52 (26.4)
One line of treatment	70 (35.5)
Two lines of treatment	32 (16.2)
Three or more lines of treatment	31 (15.7)
Unknown	12 (6.0)
**Origen of the samples**	
Primary tumor	123 (62.4)
Metastasis	73 (37.1)
Unknown	1 (0.5)

* Others include cervical (4), gastric (4), pancreas (4), melanoma (2), anal (1), appendiceal (1), esophageal (1), renal (1), oral cavity (1) and thyroid (1) cancer

### Mutational detection

**Figure 1:**
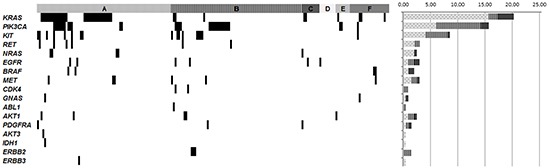
Genomic mutations found across the different solid tumor types enrolled in the study Selected genes are mutated in at least one tumor sample. Samples with mutations are shown in black. **A.** Colorectal samples; **B.** Breast cancer samples; **C.** Ovarian cancer samples; **D.** Lung cancer samples; **E.** Endometrial cancer samples, **F.** Other tumor samples (oral cavity, cervical, melanoma, gastric, anal, renal, pancreatic, appendiceal, esophageal and thyroid cancers). The histogram represents the percentage of gene mutation across the different tumor types. Colorectal cancer and breast cancer are represented with dots and with lines, respectively, while all other tumors are represented together in black.

A total of 197 samples were subjected to a hotspot mutation screening of 25 known cancer genes using the OncoCarta Panel v1.0 (Sequenom, San Diego, CA) and two customized panels. Mutations with frequencies higher than 10% were detected with high accuracy. One hundred and thirty-four oncogenic mutations were detected in 97 (49.2%) patients, and these mutations were found in the *KRAS*, *PIK3CA*, *KIT*, *MET*, *RET*, *NRAS*, *EGFR*, *BRAF*, *CDK4*, *GNAS*, *ABL1*, *AKT1*, *AKT3*, *PDGFRA*, *IDH1*, *ERBB2* and *ERBB3* genes (Figure 1 and Supplementary Figure S1). A total of 49 different oncogenic mutations were identified, 33 (80.5%) of them base transitions. The RAS/RAF/MAPK and the PIK3/AKT pathways were the most frequently mutated with 50 (51.5%) and 35 (36.1%) tumors mutated, respectively. Mutations in the *KRAS* gene were detected in 40/97 (41.2%) patients whereas mutations in the *PIK3CA* gene were detected in 30/97 (30.9%) patients. See Supplementary Table S2. Furthermore, 31 patients had mutations in at least two genes (32.0%), 2 of them carriers of synchronous mutations within the *PIK3CA* oncogene. Moreover, 3 of the samples carried more than two different mutations.

**Figure 2:**
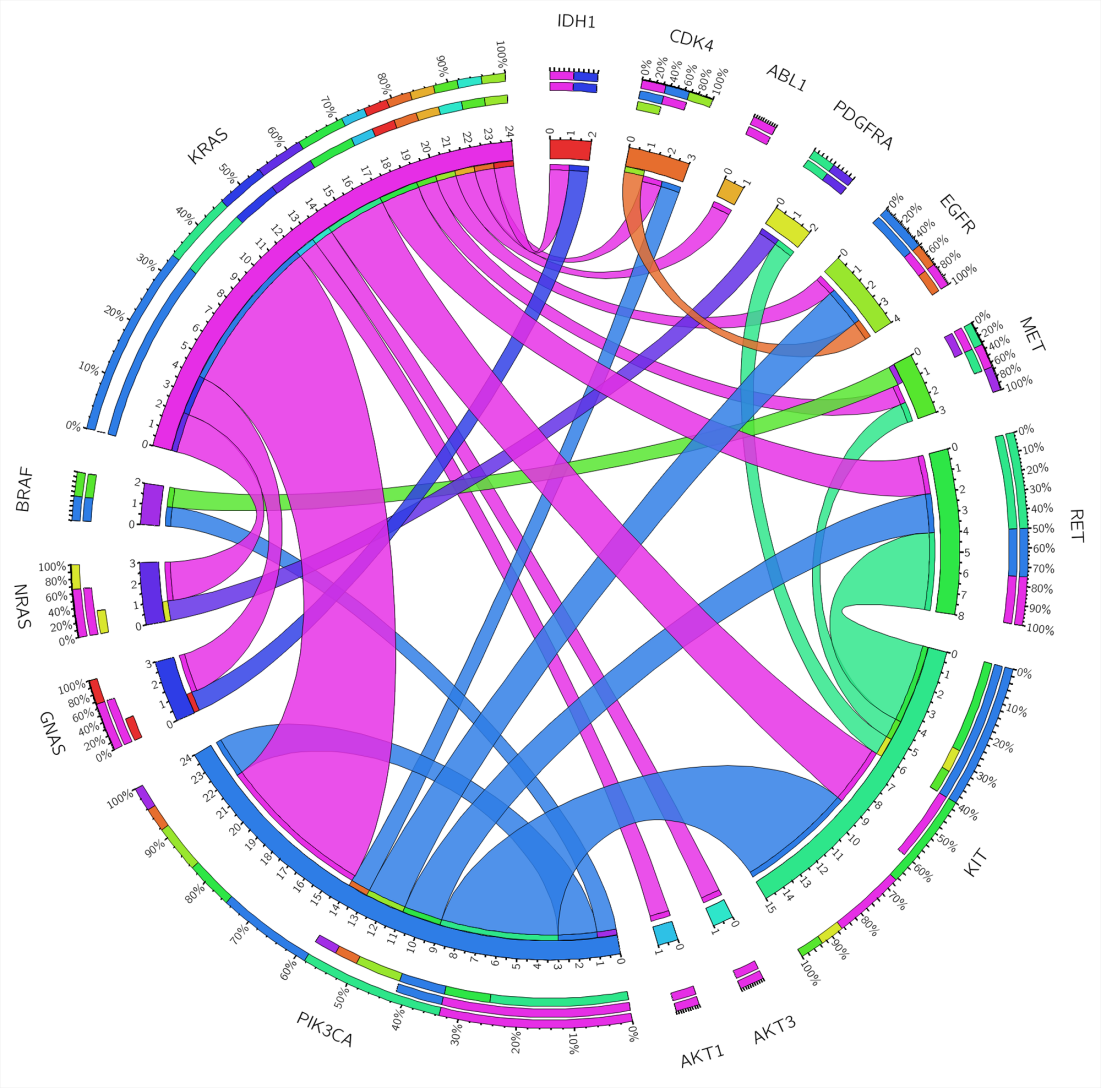
Genomic co-occurrence mutations found across those tumor samples with two or more mutations The length of the arc corresponds to the frequency of mutations in the first gene, and the width of the ribbon corresponds to the percentage of patients who also had a mutation in the second gene. This diagram was obtained using the Circos software (http://mkweb.bcgsc.ca/tableviewer/visualize/).

Twenty of the 31 cases with co-occurrence mutations (64.5%) were initially diagnosed with colorectal cancer. First, the co-occurrence of mutations within *KRAS* and *PIK3CA* was found in 8 (25.8%) patients. *KRAS* mutations were mainly located within exon 2, affecting G12 and G13 amino-acids, whereas *PIK3CA* mutations were mainly located in the helical domain, in positions 420, 452 and 546. Second, the mutations found in *KIT* and *PIK3CA* were found in 6 (19.4%) patients. These mutations affected amino-acids D52 and E839 in *KIT* and E542, E545 and H1047 in *PIK3CA*. Interestingly, mutation E839K in *KIT* appeared exclusively with the *PIK3CA* E452K mutation. Last, the co-mutations in *KIT* and *RET* were present in 4 (12.9%) patients. These mutations were D52N in the *KIT* gene and C634W in the *RET* gene (Table 2 and Figure 2).

**Table 2:** Samples with co-occurrence of mutations

Sample	Type of Cancer	Most frequently mutated genes				Least frequently mutated genes			
		*KRAS*	*PIK3CA*	*KIT*	*RET*	Gene	Mutation	Gene	Mutation
INV063	Breast, liver mets	G12D				*ABL1*	Y253H		
INV110	Rectal	G13D				*AKT1*	E17K		
INV086	Colon	G13D				*AKT3*	G171R		
INV034	Cervix	G12D				*GNAS*	R201H		
INV198	Colon	G12D				*GNAS*	R201H		
INV161	Colon	G12C				*KIT*	D52N		
INV005	Rectal, lung mets	G12D				*MET*	R970C		
INV017	Rectal	A146V				*NRAS*	G12S		
INV186	Colon	G13D				*NRAS*	G13D		
INV016	Colon	**G12D**	**E542K**	**D52N**	**C634W**				
INV163	Colon	**G13D**	**E542K & H1047R**	**D52N**					
INV042	Breast	**Q61R**	**C420R**			*CDK4*	R24C	*EGFR*	P772_H773InsV
INV028	Colon	**G12D**	**E542K**						
INV059	Colon	**G12S**	**E542K**						
INV084	Colon	**G13D**	**Q546R**						
INV181	Colon	**G12V**	**Y1021C**						
INV185	Colon	**G12C**	**G1049R**						
INV001	Colon	G12D			C634Y				
INV045	Colon		C420R			*BRAF*	V600E		
INV054	Colon		G1049R			*EGFR*	D770_N771>AGG		
INV177	Breast		**E545K**	**D52N**					
INV036	Cervix		**E542K**	**E839K**					
INV141	Colon, liver mets		**H1047R**	**D52N**	**C634W**				
INV126	Breast		**E542K**	**E839K**					
INV088	Breast, pleural mets		**E545K & G1049R**						
INV134	Breast, lung mets			L576P		*MET*	N375S		
INV055	Rectal			D52N		*PDGFRA*	D842V		
INV081	Colon			**D52N**	**C634W**				
INV071	Breast, pleural mets			**D52N**	**C634W**				
INV011	Kidney					*MET*	R970C	*BRAF*	L597S
INV023	Ovary					*NRAS*	G13D	*PDGFRA*	D1071N

Most and least frequently mutated genes in samples with co-occurrence mutations

mets: metastasis

Bold represent the most frequent associations: *KRAS* + *PIK3CA*; *PIK3CA* + *KIT* and *KIT* + *RET*

The concordance between the OncoCarta Panel v1.0 and the customized panels was 90.0%. Moreover, the concordance between the OncoCarta Panel v1.0 and Junior NGS technology was 88.0% (data not shown). In the present study, taking into account the mutations with frequencies higher than 10%, the sensibility and specificity were 79.0% and 93.5%, respectively. Those samples with non-concordant results had low allelic frequency mutations.

### Association with clinical characteristics

Association with clinical characteristics was performed for the two most represented tumor types.

#### Colorectal cancer

Overall, mutations were detected in 48 of 75 (64.2%) available FFPE tumors, predominantly in primary tumor samples (37/48, 77.1%) (Table 3 and Supplementary Table S1). Specifically, *KRAS*, *PIK3CA* and *KIT* mutations were detected in 31/48 (64.6%), 11/48 (22.9%), 8/48 (16.7%) tumor specimens, respectively. There was (66/75) 88.0% concordance for FFPE tumoral mutation status between the OncoCarta Sequenom panel and the next generation sequencing (NGS) Junior (Roche). Mutations in five of the samples found by NGS were not detected by Sequenom (*KRA*S p.G12C 10.8%, p.Q61K 47.1% and p.A146T 11.6%, and p.Q61L 13.7% and *BRAF* p.V600E 12.3%; percentages represent the frequency of mutant alleles). Four of them were close to the 10% threshold established. Among the other 4 samples, mutations were detected only by the Sequenom technology (*KRAS* p.G12D 15.0% and p.G13D 15.2%, *NRAS* p.G13D 21.4% and *PIK3CA* p.H1047R 23.0%).

**Table 3:** Mutation distribution across colorectal cancer samples

Sample	Location	Type	Gene	Mutation	%M	Gene	Mutation	%M	Gene	Mutation	%M	Gene	Mutation	%M
INV001	Left colon	Primary	*KRAS*	G12D	42.7	*RET*	C634Y	10.5						
INV004	Left colon	Primary	*KRAS*	A146V	23.3									
INV014	Left colon	Primary	*KRAS*	G12D	35.3									
INV086	Left colon	Primary	*KRAS*	G13D	39.8	*AKT3*	G171R	15.1						
INV104	Left colon	Primary	*KRAS*	G12V	31.6									
INV164	Left colon	Primary	*KRAS*	G12D	33.7									
INV060	Left colon	Metastasis	*KRAS*	Q61R	36.9									
INV008	Left colon	Primary	*PIK3CA*	G1049R	14.5									
INV054	Left colon	Primary	*PIK3CA*	G1049R	10.6	*EGFR*	D770_N771>AGG	11.1						
INV138	Left colon	Metastasis	*PIK3CA*	G1049R	10.7									
INV081	Left colon	Primary	*KIT*	D52N	27.6	*RET*	C634W	31.8						
INV161	Left colon	Primary	*KIT*	D52N	14.6	*KRAS*	G12C	24.1						
INV141	Left colon	Metastasis	*KIT*	D52N	47.2	*PIK3CA*	H1047R	23.0	*RET*	C634W	42.4			
INV154	Left colon	Metastasis	*MET*	N375S	34.2									
INV180	Left colon	Primary	*AKT1*	E17K	36.6									
INV186	Left colon	Primary	*KRAS*	G13D	15.2	*NRAS*	G13D	21.4						
INV196	Left colon	Primary	*KRAS*	G12V	14.3									
INV201	Left colon	Primary	*NRAS*	G12D	24.1									
INV016	Right colon	Primary	*KRAS*	G12D	10.0	*KIT*	D52N	10.4	*PIK3CA*	E542K	13.4	*RET*	C634W	23.5
INV026	Right colon	Primary	*KRAS*	G12D	35.0									
INV028	Right colon	Primary	*KRAS*	G12D	14.0	*PIK3CA*	E542K	14.4						
INV031	Right colon	Primary	*KRAS*	G12D	38.6									
INV059	Right colon	Primary	*KRAS*	G12S	23.4	*PIK3CA*	E542K	19.4						
INV066	Right colon	Primary	*KRAS*	G13D	40.9									
INV084	Right colon	Primary	*KRAS*	G13D	24.6	*PIK3CA*	Q546R	12.7						
INV163	Right colon	Primary	*KRAS*	G13D	27.0	*KIT*	D52N	20.0	*PIK3CA*	E542K	14.6	*PIK3CA*	H1047R	23.1
INV082	Right colon	Metastasis	*KRAS*	A59T	19.5									
INV045	Right colon	Primary	*BRAF*	V600E	15.7	*PIK3CA*	C420R	23.3						
INV181	Right colon	Primary	*KRAS*	G12V	31.1	*PIK3CA*	Y1021C	72.0						
INV185	Right colon	Primary	*KRAS*	G12C	15.0	*PIK3CA*	G1049R	15.0						
INV193	Right colon	Primary	*KRAS*	G12S	26.9									
INV197	Right colon	Primary	*BRAF*	V600E	11.5									
INV198	Right colon	Primary	*KRAS*	G12D	67.5	*GNAS*	R201H	31.1	*IDH1*	R132C	44			
INV017	Rectum	Primary	*KRAS*	A146V	10.8	*NRAS*	G12S	46.0						
INV110	Rectum	Primary	*KRAS*	G13D	36.5	*AKT1*	E17K	33.9						
INV005	Rectum	Metastasis	*KRAS*	G12D	49.4	*MET*	R970C	48.9						
INV018	Rectum	Primary	*NRAS*	Q61R	23.8									
INV020	Rectum	Primary	*KIT*	D52N	19.8									
INV055	Rectum	Primary	*KIT*	D52N	27.8	*PDGFRA*	D842V	20.5						
INV030	Rectum	Metastasis	*KIT*	D52N	*16.4*									
INV147	Rectum	Primary	*EGFR*	G719S	*22.7*									
INV079	Unknown	Primary	*KRAS*	G12D	*58.3*									
INV145	Unknown	Primary	*KRAS*	A146T	*16.7*									
INV184	Unknown	Primary	*MET*	N375S	*27.5*									
INV190	Unknown	Metastasis	*KRAS*	G12C	*33.4*									
INV191	Unknown	-	*KRAS*	G12V	*15.4*									

%M represent the percentage of mutant alleles in each reported gene

#### Breast cancer

Overall, mutations were detected in 34 of 73 (46.6%) available FFPE tumors, predominantly in the metastatic tissue (23/34, 67.6%) (See Table 4 and Supplementary Table S1). Specifically, *PIK3CA* and *KIT* mutations were detected in 16 (47.0%), and 8 (23.5%), respectively, of the tumor specimens. There was (70/73) 96.0% concordance for FFPE tumoral mutation status between the OncoCarta Sequenom panel and the NGS Junior. Two samples showed *PIK3CA* mutations in NGS but not in Sequenom (p.E542K, 29% and p.H1047L 11%; percentages represent the frequency of mutant alleles). The last reported mutation is close to the threshold of detection by Sequenom technology. Finally, the last, fourth, sample showed *AKT1* mutation in NGS, but not in Sequenom (p.E17K 44.8%; percentage represents the frequency of mutant alleles).

**Table 4:** Mutation distribution across breast cancer samples

Sample	Molecular subtype	Histology	Type	Gene	Mut	%M	Gene	Mut	%M	Gene	Mut	%M	Gene	Mut	%M
INV174	Luminal A	Ductal	Primary	*KIT*	D52N	*15.3*									
INV096	Luminal A	Ductal	Metastasis	*KIT*	D52N	*23.8*									
INV095	Luminal A	Ductal	Metastasis	*KIT*	K550_K558del	*17.6*									
INV107	Luminal A	Lobular	Metastasis	*PIK3CA*	N345K	*12.5*									
INV033	Luminal A	Ductal	Metastasis	*PIK3CA*	E542K	*32.0*									
INV117	Luminal A	Ductal	Metastasis	*PIK3CA*	M1043I	*15.5*									
INV072	Luminal A	Lobular	Metastasis	*PIK3CA*	H1047R	*15.8*									
INV205	Luminal A	Ductal	Metastasis	*PIK3CA*	H1047R	*37.7*									
INV169	Luminal B	Ductal	Primary	*AKT1*	E17K	*20.4*									
INV170	Luminal B	Ductal	Metastasis	*AKT1*	E17K	*59.9*									
INV128	Luminal B	Ductal	Metastasis	*EGFR*	H773_V774insH	*30.9*									
INV071	Luminal B	Lobular	Metastasis	*KIT*	D52N	*14.3*	*RET*	C634W	48.3						
INV073	Luminal B	Ductal	Metastasis	*KIT*	Y553_Q556del	*11.3*									
INV155	Luminal B	Not specified	Primary	*MET*	N375S	*35.1*									
INV173	Luminal B	Ductal	Metastasis	*PIK3CA*	E542K	*37.8*									
INV126	Luminal B	Lobular	Primary	*PIK3CA*	E542K	*12.0*	*KIT*	E839K	14.4						
INV105	Luminal B	Ductal	Metastasis	*PIK3CA*	E545K	*57.4*									
INV177	Luminal B	Ductal	Primary	*PIK3CA*	E545K	*59.3*	*KIT*	D52N	10.0						
INV077	Luminal B	Ductal	Metastasis	*PIK3CA*	E545K	*30.4*									
INV088	Luminal B	Ductal	Metastasis	*PIK3CA*	E545K	*66.3*	*PIK3CA*	G1049R	19.2						
INV101	Luminal B	Ductal	Primary	*PIK3CA*	H1047R	*20.5*									
INV092	Luminal B	Ductal	Primary	*RET*	C634W	*31.3*									
INV042	Basal like	Ductal	Primary	*PIK3CA*	C420R	*12.1*	CDK4	R24C	13.2	*EGFR*	P772_H773insV	*13.2*	*KRAS*	Q61R	12.4
INV057	Basal like	Ductal	Primary	*PIK3CA*	H1047R	*18.9*									
INV044	Basal like	Lobular	Metastasis	*PIK3CA*	H1047R	*19.5*									
INV069	Her2	Ductal	Metastasis	*PIK3CA*	M1043I	*49.6*									
INV074	Her2	Tubule-lobular	Metastasis	*PDGFRA*	D842V	*32.2*									
INV094	Her2	Ductal	Primary	*KRAS*	G12D	*14.8*									
INV134	Her2	Ductal	Metastasis	*KIT*	L576P	*13.3*	*MET*	N375S	17.6						
INV070	Her2	Ductal	Metastasis	*CDK4*	R24H	*10.4*									
INV063	Her2	Ductal	Metastasis	*ABL1*	Y253H	*12.2*	*KRAS*	G12D	22.6						

Mut, mutation; %M represent the percentage of mutant alleles in each reported gene

### Personalized therapy

A total of 101 patients could benefit from targeted therapies. Seventy-five of the patients presented potential actionable mutations, whereas an additional 26 patients with colorectal cancer had *KRAS* wild type status. Among these 101 patients, 28 received genotype-directed therapy (28.0%), including 20 colorectal cancer patients that received clinically available agents. Five of these 20 colorectal cancer patients (25.0%) received anti-EGFR therapy, whereas the rest (15 patients) received other available therapies.

The remaining 8 patients were enrolled in clinical trials. These patients had breast or gynecological malignancies. Seven of them carried a mutation in the *PI3KCA* gene, and one had a mutation in the *ERBB2* gene. Among the *PI3KCA* mutation carriers, 5 received PI3K/AKT inhibitors. The other 3 received other target drugs, including an anti-IGF1 therapy in one case and an anti-ERBB3 therapy in two patients (See Supplementary Figure S2).

A total of 73 patients who could possibly have benefitted from targeted therapies were not treated. The most common reasons for not offering targeted therapies according to the mutations found were diverse. Consequently, twenty-five patients (34.3%) followed standard therapies. Another 19 (26%) patients did not progress during the study period and did not require a new treatment. The rest 29 (39.7%) were not eligible due to co-morbidities, poor performance status, concurrent secondary neoplasm or loss of follow up.

## DISCUSSION

Many different solid tumors contain hotspot mutations within oncogenes that confer a relevant susceptibility or resistance to targeted anticancer therapies. A comprehensive characterization of several cancer genomes has been made possible as a result of the development of NGS technologies. At present, however, these techniques are still not fully cost-effective for the medium-sized clinical laboratory. The analysis of key cancer-driving mutations using mass-spectrometry is a cost-effective, sensitive high throughput approach for identifying mutations of clinical relevance to molecular-based therapy [17].

Sequenom technology has been recently approved for clinical diagnosis, allowing mutation frequencies of as low as 1% to be detected. Although in the present study, mutations with frequencies higher than 10% were considered to be positive, samples were deeply evaluated for their tumor content, and only sections containing more than 30% tumor cells were considered in order to detect targetable aberrations. This threshold percentage was established by others as an accurate and detectable level of rare alleles [17–19]. The present approach focused only on oncogenes hotspots and did not contemplate other mutations or tumor suppressors. Furthermore, the infrequent variations might not have any association with therapy. Therefore, this methodology makes it possible for a medium-sized laboratory to analyse multiple key hotspot mutations rapidly (within 3 days) and without complex bioinformatics analysis tools at a moderate price. At present, NGS technology is becoming more accessible, and the analysis is being simplified. Sequenom technology, however, remains a good validation technology and is optimal when only hotspots are pursued.

In the present study, we have characterized the mutation status of 25 known cancer genes in a large series of 197 solid tumors from various anatomical sites using the Sequenom Platform. The mutation sites included in the Sequenom OncoCarta Panel v1.0 assay are frequently seen in many different types of solid tumors and are clinically actionable. Mutations in 17 different genes at 49 different nucleotide positions were detected in 97 of our cancer patients, of which 28 received targeted therapies. Thus, the overall rate of success in matching patients to personalized treatments was 28 out of 97 (28.0%), similar to other recently published studies [20–22]. This rate includes 20 CRC samples treated both by anti-EGFR, as well as other available therapies. The remaining 8 patients treated were enrolled in clinical trials, most of them against PI3K/AKT inhibitors, in accordance with other publications [23]. In the present series, the *KRAS* and the *PIK3CA* genes were the most frequently mutated genes in 41.2% and 30.9% of the mutated patients, respectively. Mutations in these genes disrupt many different and overlapping signaling pathways, including the PI3K/AKT and ERK/MAPK, influencing important cellular processes. Cross-validation of detected mutations was feasible by two customized mass-spectrometry panels and NGS Junior 454 Roche technology with a concordance rate of 90.0% and 88.0%, respectively. Concordance was considered when the same alleles at similar mutation frequencies were detected by the two different panels or techniques. MassARRAY technology's high sensibility and specificity made the results obtained with this platform highly reproducible.

Colorectal and breast cancer were the two most represented tumor types with 75 and 73 cases enrolled, respectively. Among colorectal cancer samples, mutations were detected in 64.0% of the analyzed tumors, a similar ratio to those previously published [17, 24–26]. In the colorectal cancer set, *KRAS* (42.5%), *PIK3CA* (17.8%) and *KIT* (10.9%) were the most frequently mutated genes. Frequencies for both *KRAS* and *PIK3CA* were similar to the COSMIC database and to those of other publications (http://cancer.sanger.ac.uk/cosmic and http://www.cbioportal.org/) (See Supplementary Table S3) [17, 24, 27]. Furthermore, sporadic mutations appeared across *RET*, *BRAF*, *EGFR*, *AKT1*, *AKT3*, *MET*, *NRAS*, *PDGFRA*, *IDH1* and *ERBB3* [24].

Among breast cancer samples, mutations were detected in 46.6% of the analyzed tumors, specifically in *PIK3CA* and *KIT*. Mutations among other genes were present in less than 5%, a rate similar to those of the COSMIC database and other studies such as The Cancer Genome Atlas Network (See Supplementary Table S3) [21, 28–30]. *PIK3CA* mutations were found in 7 (46.7%) luminal B, 4 (26.7%) luminal A, 3 (20.0%) basal-like and 1 (6.7%) HER2 subtypes (Breast cancer subtypes according to Perou and colleagues, 2000) [31]. Nevertheless, half of all the HER2 subtype tumors carried at least one mutation, and *PIK3CA* mutations were more frequently found in estrogen receptor-positive cancers compared to triple negative breast cancer [28].

At present, KIT mutations are without clinical implications in the current therapeutical approach to colorectal and breast cancer.

The present work focused on individuals with advanced solid tumors and potential candidates to phases I/II clinical trials due to initial treatment failure. Variations in frequencies between our data and other reports may be attributed to advanced tumor selection and the number of samples analyzed.

Interestingly, one third of the patients with mutated tumors had two genes altered, of which two thirds were initially diagnosed as colorectal cancer. Two patients carried synchronous mutations within the *PIK3CA* oncogene. Among breast cancer samples, co-occurrence appeared mainly in *PIK3CA* and *KIT*. In the colorectal cancer cases, however, co-mutation was observed most frequently in the *KRAS* and *PIK3CA* genes. The *KRAS*, *NRAS* and *BRAF* mutations in colorectal cancer are normally mutually exclusive. Conversely, the coexistence of mutations in *KRAS* and *PIK3CA* has been described in a significant percentage of colorectal tumors, confirming the parallel activation of ERK/MAPK and PI3K/AKT signaling convergent pathways [15, 32].

Remarkably, the co-occurrence of mutations within *KRAS* and *PIK3CA* was the most common, in 8 (25.8%) patients. *KRAS* mutations were mainly located within exon 2, affecting the functionally G12 and G13 amino-acids. Co-existent *PIK3CA* mutations were mainly located in the helical domain, in positions 420, 452 and 546. The coexistence of *PIK3CA* and *KRAS* mutations has been shown in several different tumors types including lung, colorectal, pancreatic and ovarian cancer [33–35].

Mutations found in *KIT* and *PIK3CA* were found in 6 (19.4%) patients, having an effect on amino-acids D52 and E839 in *KIT* and E542, E545 and H1047 in *PIK3CA*. Interestingly, mutation E839K in *KIT* appeared exclusively with the *PIK3CA* E452K mutation. Finally, co-mutations in *KIT* and *RET* were present in 4 (12.9%) patients. These mutations were D52N in the *KIT* gene and C634W in the *RET* gene. The co-occurrence of mutations in *KIT* and *PIK3CA* or *RET* has been described very little. Results obtained from The Cancer Genome Atlas Network for both colorectal and breast cancer showed the co-existence of mutations in these genes, although in low proportions (4.93% for *PIK3CA* and *KIT* and 1.23% for *KIT* and *RET*).

These facts suggest that cancer development may progress due to accumulation of different somatic driver mutations, affecting different pathways. At the same time, the presence of several mutations across different genes may point out tumor heterogeneity and suggest the presence of subclones. It is the detection of different clones, some of which may show resistance to therapies, a major concern, that is changing standard therapeutic approaches.

The present study aimed at identifying key alterations that may represent important targets for novel therapies. We used mass-spectrometry, an effective and high throughput approach, which successfully detected frequent cancer mutations in degraded DNA isolated from FFPE samples and provided some advantages in terms of minimizing cost and time. This technology, in combination with the OncoCarta Panel v1.0, covers up to 95% of known druggable markers for an efficient mutation screening in clinical research trials and has an elevated grade of concordance with NGS technologies.

## MATERIALS AND METHODS

### Patient selection and data collection

The design of the study was exploratory and prospective. A total of 213 consecutive and non-related cancer cases were recruited from September 2013 to December 2014 at the Hematology and Medical Oncology Unit of the Clinic University Hospital in Valencia, Spain. Patient eligibility criteria included clinical and histological diagnoses of advanced solid cancer or potential candidates to phases I/II clinical trials due to initial treatment failure and at least one biopsiable lesion.

Clinical information, including age, sex, tumor type, location and treatments were collected (See Table 1). All study subjects gave written, informed consent, and the study was approved by the Biomedical Research Institute INCLIVA Ethics Committee.

Formalin-fixed paraffin-embedded (FFPE) tissues were evaluated for their tumor content, and sections containing more than 30% tumor cells were defined and cut by an expert pathologist. Genomic DNA was isolated from 4 unstained sections of 20 μm and diluted to a final solution of 10ng/μl. This was done using two extraction kits: Recover All Total Nucleic Acid Isolation kit (Ambiom, Life Technologies) and the QIAamp DNA FFPE tissue kit (QIAGEN). DNA concentration was quantified in samples by NanoDrop (NanoDrop Technologies, Wilmington, DE, USA).

Sixteen cases did not yield DNA of sufficient quantity, and were excluded from further analyses, leaving 197 samples in the study.

### Sequenom MassARRAY somatic mutation genotyping

The Sequenom MassARRAY and OncoCarta Panel v1.0 were used following the manufacturer's protocol (Sequenom, San Diego, CA, USA; (http://agenabio.com/oncocarta-panel)). The panel consisted of 24 multiplex assays capable of detecting 238 mutations in 19 oncogenes. This procedure was a rapid, cost-effective method of identifying key cancer driving mutations across a large number of samples because it avoided complex bioinformatic analyses and assays were performed within two days. The amount of DNA added to the polymerase chain reaction was 20 ng per reaction. DNA was amplified using the OncoCarta PCR primer pools. Unincorporated nucleotides were inactivated by shrimp alkaline phosphatase (SAP), and a single base extension reaction was performed using extension primers that hybridize immediately adjacent to the mutations and a custom mixture of nucleotides. Salts were removed by the addition of a cation exchange resin. Multiplexed reactions were spotted onto SpectroCHIP II arrays, and DNA fragments were resolved by MALDI-TOF on the Compact Mass Spectrometer (Sequenom, San Diego, CA). Two additional customized mutation panels were used. These panels were designed in collaboration with the Cancer Genomics Group at the Vall d'Hebron Institute of Oncology and included, in 12 multiplexes, a total of 107 somatic mutations in 15 genes. These two panels included 49 additional positions in 6 additional genes. Therefore, a total of 287 different positions in 25 oncogenes were checked (See Supplementary Table S4).

### Next generation sequencing (NGS)

The Junior 454 Roche sequencing technology was used by the Genotyping and Genetic Diagnosis Unit (UCIM) following the manufacturer's protocol. This sequencing technology was used to analyze hotspot mutations in the *AKT1*, *BRAF*, *EGFR*, *KRAS*, *NRAS* and *PIK3CA* genes. A complete list of all the informed mutations is provided in Supplementary Table S5.

### Statistical analyses

Data were analyzed using the Sequenom MassARRAY Typer Analyser 4.0 Software to visualize the mass spectra for mutations and to determine the frequency of mutant and wild-type alleles. The lower threshold for mutation detection has been between 5-10% [17–19]. In order to reduce putative false positives we set the threshold at 10%. More specifically, only mutations with frequencies higher than 10% were taken as positive results. Mutations were manually reviewed by use of visual and raw spectrum patterns. Two different personnel in the laboratory scored mutations, and no discrepancies were observed. Analyses were performed using IBM SPSS Statistics for Windows, Version 19.0. Armonk, NY: IBM Corp (IBM Corp. Released 2010).

## SUPPLEMENTARY FIGURES AND TABLES



## References

[1] Dancey JE, Bedard PL, Onetto N, Hudson TJ (2012). The genetic basis for cancer treatment decisions. Cell.

[2] Vucic EA, Thu KL, Robison K, Rybaczyk LA, Chari R, Alvarez CE, Lam WL (2012). Translating cancer ‘omics’ to improved outcomes. Genome Res.

[3] Chapman PB, Hauschild A, Robert C, Haanen JB, Ascierto P, Larkin J, Dummer R, Garbe C, Testori A, Maio M, Hogg D, Lorigan P, Lebbe C (2011). Improved survival with vemurafenib in melanoma with BRAF V600E mutation. N Engl J Med.

[4] Yamaguchi M, Harada K, Ando N, Kawamura T, Shibagaki N, Shimada S (2011). Marked response to imatinib mesylate in metastatic acral lentiginous melanoma on the thumb. Clin Exp Dermatol.

[5] Costa C, Molina MA, Drozdowskyj A, Gimenez-Capitan A, Bertran-Alamillo J, Karachaliou N, Gervais R, Massuti B, Wei J, Moran T, Majem M, Felip E, Carcereny E (2014). The impact of EGFR T790M mutations and BIM mRNA expression on outcome in patients with EGFR-mutant NSCLC treated with erlotinib or chemotherapy in the randomized phase III EURTAC trial. Clin Cancer Res.

[6] Rosell R, Moran T, Queralt C, Porta R, Cardenal F, Camps C, Majem M, Lopez-Vivanco G, Isla D, Provencio M, Insa A, Massuti B, Gonzalez-Larriba JL (2009). Screening for epidermal growth factor receptor mutations in lung cancer. N Engl J Med.

[7] Marks JL, Broderick S, Zhou Q, Chitale D, Li AR, Zakowski MF, Kris MG, Rusch VW, Azzoli CG, Seshan VE, Ladanyi M, Pao W (2008). Prognostic and therapeutic implications of EGFR and KRAS mutations in resected lung adenocarcinoma. J Thorac Oncol.

[8] Cunningham D, Humblet Y, Siena S, Khayat D, Bleiberg H, Santoro A, Bets D, Mueser M, Harstrick A, Verslype C, Chau I, Van Cutsem E (2004). Cetuximab monotherapy and cetuximab plus irinotecan in irinotecan-refractory metastatic colorectal cancer. N Engl J Med.

[9] Van Cutsem E, Peeters M, Siena S, Humblet Y, Hendlisz A, Neyns B, Canon JL, Van Laethem JL, Maurel J, Richardson G, Wolf M, Amado RG (2007). Open-label phase III trial of panitumumab plus best supportive care compared with best supportive care alone in patients with chemotherapy-refractory metastatic colorectal cancer. J Clin Oncol.

[10] Douillard JY, Oliner KS, Siena S, Tabernero J, Burkes R, Barugel M, Humblet Y, Bodoky G, Cunningham D, Jassem J, Rivera F, Kocakova I, Ruff P (2013). Panitumumab-FOLFOX4 treatment and RAS mutations in colorectal cancer. N Engl J Med.

[11] Hauschild A, Grob JJ, Demidov LV, Jouary T, Gutzmer R, Millward M, Rutkowski P, Blank CU, Miller WH, Kaempgen E, Martin-Algarra S, Karaszewska B, Mauch C (2012). Dabrafenib in BRAF-mutated metastatic melanoma: a multicentre, open-label, phase 3 randomised controlled trial. Lancet.

[12] Lievre A, Bachet JB, Le Corre D, Boige V, Landi B, Emile JF, Cote JF, Tomasic G, Penna C, Ducreux M, Rougier P, Penault-Llorca F, Laurent-Puig P (2006). KRAS mutation status is predictive of response to cetuximab therapy in colorectal cancer. Cancer Res.

[13] Long GV, Stroyakovskiy D, Gogas H, Levchenko E, de Braud F, Larkin J, Garbe C, Jouary T, Hauschild A, Grob JJ, Sileni VC, Lebbe C, Mandala M (2014). Combined BRAF and MEK Inhibition versus BRAF Inhibition Alone in Melanoma. N Engl J Med.

[14] Larkin J, Ascierto PA, Dreno B, Atkinson V, Liszkay G, Maio M, Mandala M, Demidov L, Stroyakovskiy D, Thomas L, de la Cruz-Merino L, Dutriaux C, Garbe C (2014). Combined Vemurafenib and Cobimetinib in BRAF-Mutated Melanoma. N Engl J Med.

[15] Dienstmann R, Rodon J, Barretina J, Tabernero J (2013). Genomic medicine frontier in human solid tumors: prospects and challenges. J Clin Oncol.

[16] Voss JS, Holtegaard LM, Kerr SE, Fritcher EG, Roberts LR, Gores GJ, Zhang J, Highsmith WE, Halling KC, Kipp BR (2013). Molecular profiling of cholangiocarcinoma shows potential for targeted therapy treatment decisions. Hum Pathol.

[17] Fumagalli D, Gavin PG, Taniyama Y, Kim SI, Choi HJ, Paik S, Pogue-Geile KL (2010). A rapid, sensitive, reproducible and cost-effective method for mutation profiling of colon cancer and metastatic lymph nodes. BMC Cancer.

[18] Fang DD, Zhang CC, Gu Y, Jani JP, Cao J, Tsaparikos K, Yuan J, Thiel M, Jackson-Fisher A, Zong Q, Lappin PB, Hayashi T, Schwab RB (2013). Antitumor Efficacy of the Dual PI3K/mTOR Inhibitor PF-04691502 in a Human Xenograft Tumor Model Derived from Colorectal Cancer Stem Cells Harboring a Mutation. PLoS One.

[19] Dutton-Regester K, Irwin D, Hunt P, Aoude LG, Tembe V, Pupo GM, Lanagan C, Carter CD, O'Connor L, O'Rourke M, Scolyer RA, Mann GJ, Schmidt CW (2012). A high-throughput panel for identifying clinically relevant mutation profiles in melanoma. Mol Cancer Ther.

[20] Ong M, Carreira S, Goodall J, Mateo J, Figueiredo I, Rodrigues DN, Perkins G, Seed G, Yap TA, Attard G, de Bono JS (2014). Validation and utilisation of high-coverage next-generation sequencing to deliver the pharmacological audit trail. Br J Cancer.

[21] Andre F, Bachelot T, Commo F, Campone M, Arnedos M, Dieras V, Lacroix-Triki M, Lacroix L, Cohen P, Gentien D, Adelaide J, Dalenc F, Goncalves A (2014). Comparative genomic hybridisation array and DNA sequencing to direct treatment of metastatic breast cancer: a multicentre, prospective trial (SAFIR01/UNICANCER). Lancet Oncol.

[22] Johnson DB, Dahlman KH, Knol J, Gilbert J, Puzanov I, Means-Powell J, Balko JM, Lovly CM, Murphy BA, Goff LW, Abramson VG, Crispens MA, Mayer IA (2014). Enabling a genetically informed approach to cancer medicine: a retrospective evaluation of the impact of comprehensive tumor profiling using a targeted next-generation sequencing panel. Oncologist.

[23] Meric-Bernstam F, Brusco L, Shaw K, Horombe C, Kopetz S, Davies MA, Routbort M, Piha-Paul SA, Janku F, Ueno N, Hong D, De Groot J, Ravi V (2015). Feasibility of Large-Scale Genomic Testing to Facilitate Enrollment Onto Genomically Matched Clinical Trials. J Clin Oncol.

[24] Gavin PG, Colangelo LH, Fumagalli D, Tanaka N, Remillard MY, Yothers G, Kim C, Taniyama Y, Kim SI, Choi HJ, Blackmon NL, Lipchik C, Petrelli NJ (2012). Mutation profiling and microsatellite instability in stage II and III colon cancer: an assessment of their prognostic and oxaliplatin predictive value. Clin Cancer Res.

[25] Brannon AR, Vakiani E, Sylvester BE, Scott SN, McDermott G, Shah RH, Kania K, Viale A, Oschwald DM, Vacic V, Emde AK, Cercek A, Yaeger R (2014). Comparative sequencing analysis reveals high genomic concordance between matched primary and metastatic colorectal cancer lesions. Genome Biol.

[26] (2012). Comprehensive molecular characterization of human colon and rectal cancer. Nature.

[27] Dienstmann R, Markman B, Tabernero J (2012). Application of monoclonal antibodies as cancer therapy in solid tumors. Curr Clin Pharmacol.

[28] Santarpia L, Qi Y, Stemke-Hale K, Wang B, Young EJ, Booser DJ, Holmes FA, O'shaughnessy J, Hellerstedt B, Pippen J, Vidaurre T, Gomez H, Valero V (2012). Mutation profiling identifies numerous rare drug targets and distinct mutation patterns in different clinical subtypes of breast cancers. Breast Cancer Res Treat.

[29] Stephens PJ, Tarpey PS, Davies H, Van Loo P, Greenman C, Wedge DC, Nik-Zainal S, Martin S, Varela I, Bignell GR, Yates LR, Papaemmanuil E, Beare D (2012). The landscape of cancer genes and mutational processes in breast cancer. Nature.

[30] (2012). Comprehensive molecular portraits of human breast tumours. Nature.

[31] Perou CM, Sorlie T, Eisen MB, van de Rijn M, Jeffrey SS, Rees CA, Pollack JR, Ross DT, Johnsen H, Akslen LA, Fluge O, Pergamenschikov A, Williams C (2000). Molecular portraits of human breast tumours. Nature.

[32] Misale S, Di Nicolantonio F, Sartore-Bianchi A, Siena S, Bardelli A (2014). Resistance to Anti-EGFR Therapy in Colorectal Cancer: From Heterogeneity to Convergent Evolution. Cancer Discov.

[33] Janku F, Tsimberidou AM, Garrido-Laguna I, Wang X, Luthra R, Hong DS, Naing A, Falchook GS, Moroney JW, Piha-Paul SA, Wheler JJ, Moulder SL, Fu S (2011). PIK3CA mutations in patients with advanced cancers treated with PI3K/AKT/mTOR axis inhibitors. Mol Cancer Ther.

[34] De Roock W, Claes B, Bernasconi D, De Schutter J, Biesmans B, Fountzilas G, Kalogeras KT, Kotoula V, Papamichael D, Laurent-Puig P, Penault-Llorca F, Rougier P, Vincenzi B (2010). Effects of KRAS, BRAF, NRAS, and PIK3CA mutations on the efficacy of cetuximab plus chemotherapy in chemotherapy-refractory metastatic colorectal cancer: a retrospective consortium analysis. Lancet Oncol.

[35] Chaft JE, Arcila ME, Paik PK, Lau C, Riely GJ, Pietanza MC, Zakowski MF, Rusch V, Sima CS, Ladanyi M, Kris MG (2012). Coexistence of PIK3CA and other oncogene mutations in lung adenocarcinoma-rationale for comprehensive mutation profiling. Mol Cancer Ther.

