# Supplementary material for: Determination of somatic oncogenic mutations linked to target-based therapies using MassARRAY technology
Source: Oncotarget. 2016 Mar 9;7(16):22543–55. doi: 10.18632/oncotarget.8002 (PMC5008380; doi:10.18632/oncotarget.8002)
Supplement: Supplementary file 1 [file oncotarget-07-22543-s001.pdf]

SUPPLEMENTARY FIGURES AND TABLES

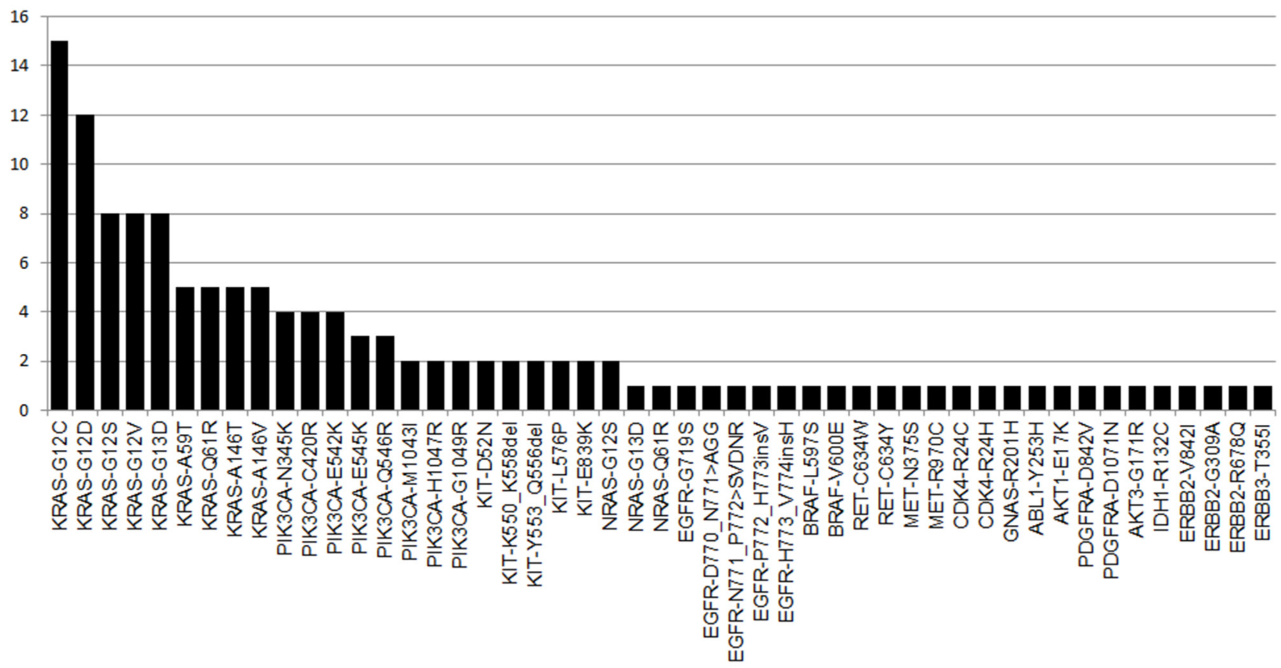

Supplementary Figure S1: Mutation frequency among all the tumor samples analyzed.

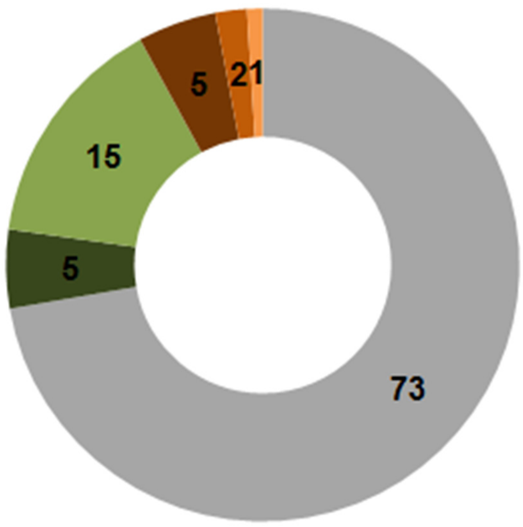

Supplementary Figure S2: Personalized therapy across all the patients enrolled in the study. Numbers represent the number of patients in each category. Possible eligible patients not treated are shown in grey. Green shades represent the number of colorectal patients treated. In dark green are those patients that received anti-EGFR whereas in light green are those patients that received other available therapies. Orange shades represent the number of patients enrolled in clinical trials. Dark orange are those patients treated with PI3K/AKT inhibitors. Medium orange and light orange represent those patients treated with anti-ERBB3 and anti-IGF1 therapy respectively.

Supplementary Table S1: Classification of colorectal and breast cancer samples by age and clinical characteristics

a. Clinical characteristics among all (N=75) and mutated (N=48) colorectal cancer samples		
**Clinical characteristic**	**Colorectal cancer set N (%)**	**Mutated colorectal cancer set N (%)**
**Age (years)**		
Median (Range)	67 (32-88)	67 (32-88)
**Gender**		
Female	24 (32.0)	17 (35.4)
Male	51 (68.0)	31 (64.6)
**Location primary tumour**		
Rectum	18 (24.0)	8 (16.7)
Right colon	21 (28.0)	16 (33.3)
Left colon	30 (40.0)	19 (39.6)
Unknown	6 (8.0)	5 (10.4)
**Histological grade**		
1	6 (8.0)	6 (12.5)
2	45 (60.0)	29 (60.4)
3	7 (9.3)	3 (6.3)
Unknown	17 (22.7)	10 (20.8)
**Prior therapy**		
No treatment	37 (49.3)	23 (47.9)
One line of treatment	26 (34.7)	15 (31.2)
Two lines of treatment	3 (4.0)	3 (6.2)
Three or more lines of treatment	6 (8.0)	5 (10.5)
Unknown	3 (4.0)	2 (4.2)
**Origen of the samples**		
Primary tumour	60 (80.0)	37 (77.1)
Metastasis sites	14 (18.7)	10 (20.8)
Unknown	1 (1.3)	1 (2.1)

b. Clinical characteristics among all (N=73) and mutated (N=34) breast cancer samples		
**Clinical characteristic**	**Breast cancer set N (%)**	**Mutated breast cancer set N (%)**
**Age (years)**		
Median (Range)	52 (29-81)	54 (31-80)
**Histological type**		
Infiltrating ductal carcinoma	59 (80.8)	25 (73.5)
Lobular	9 (12.3)	6 (17.6)
Tubule-lobular	1 (1.4)	1 (2.9)
Non specified type of carcinoma	2 (2.7)	1 (2.9)
Inflammatory	2 (2.7)	1 (2.9)

(*Continued*)

b. Clinical characteristics among all (N=73) and mutated (N=34) breast cancer samples

Clinical characteristic	Breast cancer set N (%)	Mutated breast cancer set N (%)
**Molecular subtype**		
Luminal A	21 (28.8)	8 (23.5)
Luminal B	35 (47.9)	17 (50.0)
Her2	9 (12.3)	6 (17.6)
Basal-like	6 (8.2)	3 (8.8)
Unknown	2 (2.7)	-
**Prior therapy**		
No treatment	4 (5.5)	3 (8.8)
One line of treatment	30 (41.1)	11 (32.4)
Two lines of treatment	17 (23.3)	10 (29.4)
Three or more lines of treatment	16 (21.9)	8 (23.5)
Unknown	6 (8.2)	2 (5.9)
**Origen of the samples**		
Primary tumour	25 (34.2)	11 (32.4)
Metastasis sites	48 (65.8)	23 (67.6)

All colorectal cancer samples were adenocarcinomas
Breast cancer molecular subtypes according to Perou C.M. et al. Nature. 2000; 406(6797):747-52

Supplementary Table S2: Mutation frequency across different tumour types samples included in the study

Tumour type	Samples analysed	Samples Mutated (%)
Colorectal	75	48 (64.00)
Breast	73	34 (46.58)
Ovary	10	4 (40.00)
Lung	9	1 (11.11)
Endometrium	8	4 (50.00)
Cervical	4	0 (0.00)
Gastric	4	3 (75.00)
Pancreas	4	0 (0.00)

Other tumour types: Melanoma (1 sample mutated from 2 analysed), oral cavity and renal (1 sample analysed from each type and both of them mutated), anal, appendiceal, oesophagus and thyroid (1 sample analysed and none mutated)

Supplementary Table S3: Gene mutation frequency across our study and data from different databases[¥]

Gene	Colorectal cancer samples							Breast cancer samples			
	Our study 75 cases	TCGA[1] 220 cases	MSKCC[2] 138 cases	Genentech[3] 72 cases	Cosmic Colon	Cosmic Rectum	Cosmic CRC	Our study 73 cases	TCGA[4] 507 cases	Sanger[5] 100 cases	Cosmic BC
% Mutated	**64.00**	69.00	67.00	74.00	-	-	-	**46.58**	43.00	40,00	-
KRAS	**42.47**	42.00	55.00	51.00	34.19	34.74	34.46	**4.11**	0.80	1.00	1.61
PIK3CA	**17.81**	20.00	20.00	31.00	19.88	11.75	15.81	**23.30**	35.00	30.00	26,30
KIT	**10.96**	3.00	0.00	6.00	11.94	9.72	10.83	**10.96**	1.00	0.00	1.07
RET	**5.48**	4.00	0.00	7.00	6.54	3.21	4.88	**2.74**	0.60	0.00	0.53
NRAS	**5.48**	9.00	3.00	3.00	4.72	7.49	6.11	**0.00**	0.00	0.00	0.71
EGFR	**2.74**	4.00	0.70	6.00	7.42	3.54	5.48	**2.74**	0.80	0.00	1.04
BRAF	**2.74**	9.00	4.00	8.00	17.23	4.51	10.87	**0.00**	0.60	1.00	1.22
MET	**4.11**	2.00	1.00	3.00	6.82	3.73	5.28	**2.74**	0.60	1.00	0.61
CDK4	**0.00**	1.00	0.00	1.00	0.94	0.83	0.89	**1.37**	0.00	0.00	0.20
GNAS	**1.37**	3.00	4.00	7.00	7.41	8.62	8.01	**0.00**	0.80	0.00	0.37
ABL1	**0.00**	1.00	0.00	4.00	4.19	3.73	3.96	**1.37**	0.80	0.00	0.45
AKT1	**2.74**	0.90	1.00	0.00	1.41	0.30	0.86	**2.74**	2.00	5.00	3.01
PDGFRA	**1.37**	5.00	3.00	3.00	5.41	4.23	4.82	**1.37**	0.60	3.00	0.53
AKT3	**1.37**	0.40	0.00	3.00	1.76	2.07	1.92	**0.00**	0.60	0.00	0.47
IDH1	**1.37**	1.00	0.00	0.00	4.49	1.17	2.83	**0.00**	0.20	0.00	0.35
ERBB2	**0.00**	4.00	4.00	3.00	4.11	2.84	3.48	**4.11**	1.00	1.00	1.61
ERBB3	**1.37**	6.00	6.00	8.00	4.44	2.91	3.68	**0.00**	2.00	3.00	1.20

CRC, Colorectal cancer. BC, Breast cancer
[¥]Databases: www.cbioportal.org and www.cancer.sanger.ac.uk/cosmic
[1]Comprehensive molecular characterization of human colon and rectal cancer. Cancer Genome Atlas Network. Nature, 2012
[2]Comparative sequencing analysis reveals high genomic concordance between matched primary and metastatic colorectal cancer lesions. Brannon A.R et al. Genome Biol, 2014
[3]Recurrent R-spondin fusions in colon cancer. Seshagiri S. et al. Nature, 2012
[4]Comprehensive molecular portraits of human breast tumours. Cancer Genome Atlas Network. Nature, 2012
[5]The landscape of cancer genes and mutation processes in breast cancer. Stephens P.J. et al. Nature, 2012

Supplementary Table S4: List of mutations analysed using the sequenom MassARRAY technology

**ABL1**	E17K, G173R, K179M, G250E, Q252H, Y253H, E255K/V, D276G, F311L, T315I, F317L, M351T, E355G, F359V, H396R
**AKT1**	E17K, G175R, rs11555435(V461L), rs11555431(P388T), rs11555432(L357T), rs12881616(E319G), rs11555433(V767A), rs11555436 (Q43*), rs34409589(E17del)
**AKT2**	S302G, R371H
**AKT3**	E17K, G171R
**BRAF**	K601E/N, G464V/E, G466V/G/E/R, F468C, G469S/E/A/V/R, D594V/G, F595L, G596R, L597S/R/Q/V, L597R_1790TG, T599I, V600A/D/E/G/K/L/M/R, K601N/E
**CDK4**	R24C/H

(*Continued*)

**EGFR**	R108K, T263P, A289V, G598V, E709K/H, E709A/G/V, G719S/C/A, M766_A767insAI, S768I, V769_D770insASV, V769_D770insCV, E746_T751del, IinsD770_N771>AGG/V769_D770insASV/V769_D770insASV, D770_N771insG, N771_P772>SVDNR, P772_H773insV, H773>NPY, H773_V774insNPH/H773_V774insPH/H773_V774insH, V774_C775insHV, T790 M, L858R, L861Q, L747_T750del, P ins/E746_A750del, T751A, -E746_T751del, I ins/S752_I759del, L747_E749del, A750P, E746_A750del, L747_E749del, A750P, L747_S752del, P753S, E746_T751del/V ins, L747_S752del/Q ins, E746_T751del, S752D/SNP C2255T, D770_N771>AGG/V769_D770insASV/V769_D770insASV, D770_N771insG, L747_T750del/P ins, E746_A750del, L747_T751del, E746_A750del/ V ins, S752_I759del, L858M
**ERBB2**	G309A, S310Y,H470Q, R678Q, L755P, I767M, M774_A775insYVMA, A775_G776insYVMA, G776S/G776LC, G776VC, G776VC/G776VC, P780_Y781insGSP, S779_P780insVGS, V842I
**ERBB3**	M91*, F94L,V104L/M/*, P262S/H , G284R, D297Y, E332*, T355I/A, A378P, Y464C, V528F, R667L, L783V, L792V, Q809R, S846I,E928G,T1169P, E1261A
**FGFR1**	S125L, P252T
**FGFR3**	R248C, S249C, G370C, Y373C, A391E, K650Q/E/T/M
**FLT3**	I836del, D835H/Y
**GNAQ**	Q209H/L/P/R/Y
**GNAS**	Q227L/R, R201H
**IDH1**	R132C/G/H/L/S/V
**IDH2**	R172G/K/M/S/W
**HRAS**	G12V/D, G13C/R/S/V/D, Q61H/L/R/P/K
**JAK2**	V617F
**KIT**	D52N, Y503_F504insAY, K550_K558del, M552L, W557R/W557R/W557G, V559D/V559A/V559G/I, K558_V560del, V560D/V560G, Y568D, D579del, F584S, P585P, K642E, D816V/H/Y, V825A, E839K, P551_V555del, Y553_Q556del, Y553_Q556del
**KRAS**	G12V/A/D/C/S/R/F, G13C/S/V/D, A59T, Q61E/K/L/R/P/H, A146T/P/V
**MET**	N375S, N848S, R970C, R988C, T992I, T1010I, H1112L/R/Y, H1124D, Y1230C, Y1235D, Y1248C/H, M1250T, M Y1253D, M1268T
**NRAS**	G12R/S/C/V/A/D/P/N/Y, G13R/S/C/V/A/D/N/Y, Q61E/H/K/L/P/R, A18T
**PDGFRA**	V561D, T674I, F808L, D846Y, N870S, D1071N, D842_H845del, I843_D846del, S566_E571>K, I843_S847>T, D842V
**PIK3CA**	R38H, Q60K, R88Q, E110K, N345K, S405F, E418K, C420R, P539R, E542K/Q/V/G, E453K, E545A/D/G/K/Q/V, Q546E/H/K/L/P/R, H701P, C901F, F909L, Y1021C/H/H, T1025A/S, M1043I/V, A1046V, H1047R/L/Y, G1049R/S,
**RET**	C634R/W/Y, E632_L633del, M918T, A664D

Sequenom assays, perform at the Biomedical Research Institute – INCLIVA, rapidly confirm hotspot mutations in key oncogenes with the highly robust MassARRAY® System. Each assay is based in an extension of a specific probe.

In our group we have three different panels:

A. A ready-to-use panel for 238 mutations in 19 oncogenes (Oncocarta™ version 1.0) designed by the Sequenom Company. Mutations included in this panel are coloured in black.

B. A first customized panel designed by Dr. Vivancos at the Cancer Genomics Group, Vall d'Hebron Institute of Oncology (VHIO), Spain. This customized panel includes 136 mutations in 13 oncogenes which are coloured in blue.

C. A second customized panel designed at Biomedical Research Institute - INCLIVA that includes 29 additional mutations in ERBB2 and ERBB3 genes (mutations in green).

These three panels in combination analyse 287 different positions in 25 oncogenes. Some of the mutations (coloured in red) are present in two of the panels and are used as internal controls.

Supplementary Table S5: List of mutations informed using the Junior 454 (Roche) next generation technology

**AKT1**	E17K
**BRAF**	V600A/D/E/G/K/L/M/R
**EGFR**	All insertions and deletions described in the literature and G719S/C/A, L858M
**KRAS**	G12V/A/D/C/S/R/F, G13C/S/V/D, Q61E/K/L/R/P/H, A146T/P/V
**NRAS**	G12R/S/C/V/A/D/P/N/Y, G13R/S/C/V/A/D/N/Y, Q61E/H/K/L/P/R
**PIK3CA**	E542K/Q/V/G, E545A/D/G/K/Q/V, H1047R/L/Y
